# The Neural Basis of Typewriting: A Functional MRI Study

**DOI:** 10.1371/journal.pone.0134131

**Published:** 2015-07-28

**Authors:** Yuichi Higashiyama, Katsuhiko Takeda, Yoshiaki Someya, Yoshiyuki Kuroiwa, Fumiaki Tanaka

**Affiliations:** 1 Department of Neurology and Stroke Medicine, Yokohama City University Graduate School of Medicine, Yokohama, Japan; 2 Department of Neurology, International University of Health and Welfare, Mita Hospital, Tokyo, Japan; 3 Center for Advanced Research for Logic and Sensibility, Keio University, Tokyo, Japan; University of Copenhagen, DENMARK

## Abstract

To investigate the neural substrate of typewriting Japanese words and to detect the difference between the neural substrate of typewriting and handwriting, we conducted a functional magnetic resonance imaging (fMRI) study in 16 healthy volunteers. All subjects were skillful touch typists and performed five tasks: a typing task, a writing task, a reading task, and two control tasks. Three brain regions were activated during both the typing and the writing tasks: the left superior parietal lobule, the left supramarginal gyrus, and the left premotor cortex close to Exner’s area. Although typing and writing involved common brain regions, direct comparison between the typing and the writing task revealed greater left posteromedial intraparietal cortex activation in the typing task. In addition, activity in the left premotor cortex was more rostral in the typing task than in the writing task. These findings suggest that, although the brain circuits involved in Japanese typewriting are almost the same as those involved in handwriting, there are brain regions that are specific for typewriting.

## Introduction

Use of a personal computer is common in everyday life and, accordingly, typing on a keyboard has become common for many people as a replacement for writing on paper. Recently, stroke patients with isolated typing impairment without aphasia, apraxia, or visuospatial impairment and with relative preservation of writing ability have been reported, and this phenomenon has been termed dystypia [1]. The patient reported by Otsuki et al. had a lesion in the left frontal lobe involving the foot of the second frontal convolution and frontal operculum [1]. Ryu et al. reported a 64-year-old right-handed man with acute infarcts in the bilateral border-zone regions, predominantly the left frontal subcortical area, who developed a sudden typing disturbance without aphasia or neglect [2]. In addition, Cooks et al. reported a 68-year-old patient with Parkinson’s disease who had a stroke in the left temporoparietal cortex and exhibited disproportionately affected typing relative to handwriting [3]. However, it is still unknown what brain lesions are crucial for dystypia because the reported cases had multiple lesions and the locations of the lesions differed across cases.

Although there has been a great amount of functional neuroimaging research to identify the neural basis of language comprehension (i.e., reading) and written language production (i.e., spelling and writing) [4–7], there have been very few neuroimaging studies of typewriting. Gordon et al. reported activation of the superior parietal lobule (SPL) in a functional magnetic resonance imaging (fMRI) study of the production of typing movements [8], although they regarded typing as a learned finger movement and focused on the motor execution rather than the literal aspect of typewriting. Purcell et al. used fMRI to investigate the neural basis of spelling via keyboard typing and found that typed spelling activated a predominantly left hemisphere network including the inferior frontal gyrus (IFG), middle frontal gyrus (MFG)/superior frontal gyrus (SFG), supramarginal gyrus (SMG), SPL, and fusiform gyrus, in common with the results from lesion studies in agraphia and functional imaging studies of writing [5]. Although overlaps in the typing and writing networks were suggested in their work, they did not compare the network for typing with that for writing in the same subjects; hence, it remains unclear whether there is a practical difference between them or not.

**Fig 1:**
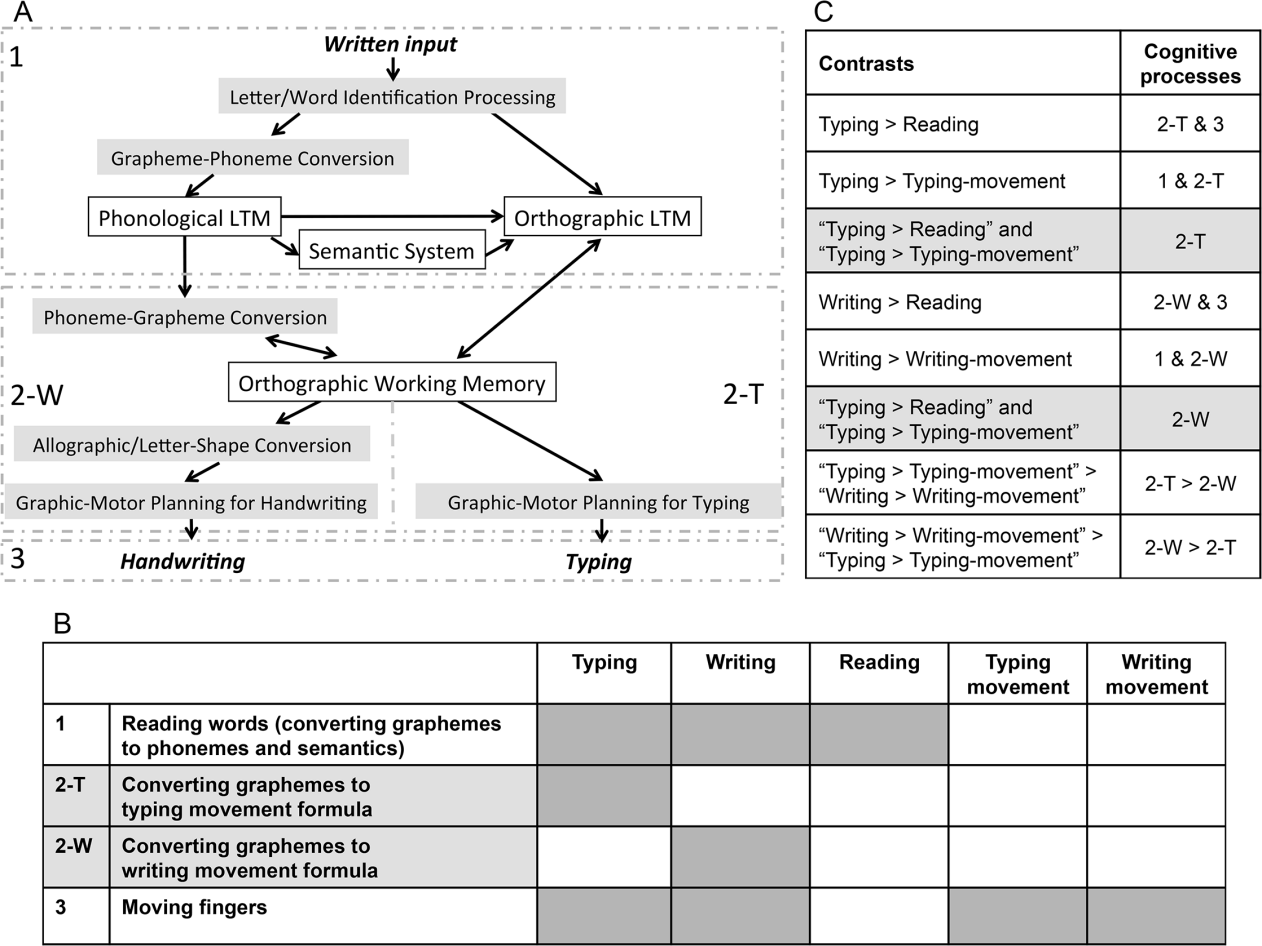
A diagram of the assumptions underlying the present analysis. (A) The cognitive model of handwriting and typing used in the present study. The labels 1, 2-W, 2-T, and 3 correspond to the labels in Fig 1B. (B) The simplified cognitive processes of typing and writing used in the present analysis. (C) The contrasts and corresponding cognitive processes. The labels 1, 2-W, 2-T, and 3 correspond to the labels in Fig 1B. Grey shading indicates conjunction contrasts.

Typing and writing words involves several cognitive processes that have been described in various cognitive models [7, 9, 10], including: phonological long-term memory (LTM), orthographic LTM, the semantic system, phoneme-grapheme conversion, orthographic working memory (graphemic buffer), and graphic-motor planning processes (Fig 1A). For example, reading involves phonological LTM, orthographic LTM, and the semantic system. Additionally, writing involves graphic-motor planning and handwriting motor commands. Although it is supposed that reading, writing, and typing share phoneme-grapheme conversion and orthographic working memory, there may be greater demands on these processes in writing and typing than in reading. We hypothesize that if there is a difference between the neural basis of typing and writing, it might be located in the transition process (orthographic working memory or graphic-motor planning) and the motoric process, because typing and writing are thought to share the same literal central processes, i.e., the same phonological LTM, orthographic LTM, semantic system, and phoneme-grapheme conversion. However, the transition process and the motoric process are quite different between these two modalities. For example, writing requires the formation of individual letters, which can involve multiple strokes per letter. By contrast, typing is more rigid with regards to form, except with capitals. Furthermore, typing is thought to involve more parallel processing, such that prior to the execution of one letter stroke, the next letter is already prepped to be executed [11]. Therefore, the activation of graphic-motor planning and the graphemic buffer related to the grapheme-motor command conversion process should differ between these two modalities.

For the purpose of elucidating the difference between typing and writing processes, we simplified the typing model by identifying three major components: (1) reading words (i.e., converting graphemes to phonemes and semantics), (2) converting graphemes to a typing movement and planning to type the corresponding keys, and (3) moving the fingers to type the keys (Fig 1B). The writing model can similarly consist of three cognitive processes: (1) reading words, (2) converting graphemes to a writing movement and planning to write the corresponding letters, and (3) moving the hand to write the letters.

The purpose of this study was to clarify the neural processes for step (2) in typing and writing. For this purpose we performed fMRI in normal volunteers performing a typewriting task that involved typing visually presented Japanese words on an MRI-compatible QWERTY keyboard and a writing task, and directly compared the brain activity recorded in these two tasks. We utilized a subtractive approach with an underlying simplistic model using fMRI. To date, there have been a very small number of brain imaging studies of writing and typing, and this is the first study to directly compare typewriting and handwriting in the same experiment.

Unlike most languages that are written using a single script, there are two writing systems in the Japanese language: kana (phonogram) and kanji (morphogram). Kana characters represent vowels or combinations of consonants and vowels, such as “ka”, “ki”, “ku”, “ke”, and “ko”, therefore, phoneme-grapheme conversion is simpler than that of alphabetic words and Japanese kana writing does not demand knowledge of spelling. By contrast, kanji can be composed of one or more characters and there are many homonyms. We employed kana script in present study because our aim was not to elucidate the literal central processes that contain semantic and lexical processes, but the peripheral processes involved in the conversion of graphemes to the typing or writing movement (graphemic buffer and graphic-motor planning process, i.e., process (2) in Fig 1A). We employed visual word stimuli to further reduce the load of semantic and lexical processes.

## Methods

### Subjects

Sixteen healthy volunteers (nine males and seven females, age 23–34 years, mean = 27.8 years, standard deviation (SD) = 3.1 years) who could touch type were recruited. All subjects were native Japanese speakers with no history of neurological or learning disorders. The Edinburgh Handedness Inventory laterality quotients [12] ranged from 88 to 100 (mean = 99.3, SD = 2.9), indicating a strong right-hand preference in all subjects. The history of PC use ranged from 8 to 25 years (mean = 14.0 years, SD = 4.5 years) and the history of touch typing ranged from 4 to 23 years (mean = 10.4 years, SD = 5.0 years), indicating that all subjects were skillful typists. All subjects had normal vision and were able to type on a Japanese QWERTY keyboard at a rate of at least 150 characters per minute without looking at their hands (mean = 333 characters per minute, SD = 65.1 characters per minute). This typing speed was assessed in a preliminary test in which subjects were asked to type a short story called “The North Wind and the Sun” from Aesop’s Fables.

All participants were recruited from students and clerks at Yokohama City University and International University of Health and Welfare. The Research Ethics Committees of the International University of Health and Welfare, Mita Hospital, and Keio University approved the experimental procedures. Written informed consent was obtained from all subjects prior to the experiment.

### Tasks and stimuli

**Fig 2:**
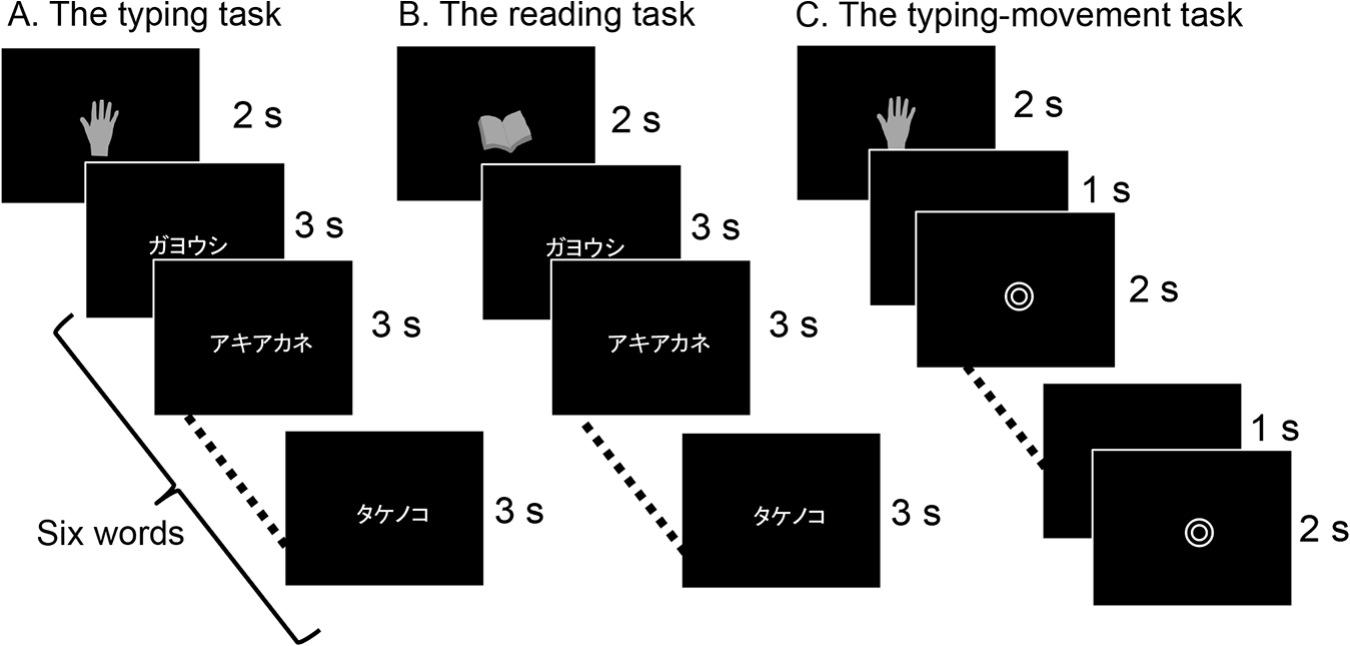
Design of the typing experiment. The typing experiment used an fMRI block design. Each session consisted of three different tasks presented pseudo-randomly in five blocks of 20-s duration. All tasks had a fixation condition. In the typing-movement task (C), subjects were instructed to type randomly with both hands when the double circle symbol was present on the monitor. fMRI: functional magnetic resonance imaging.

**Fig 3:**
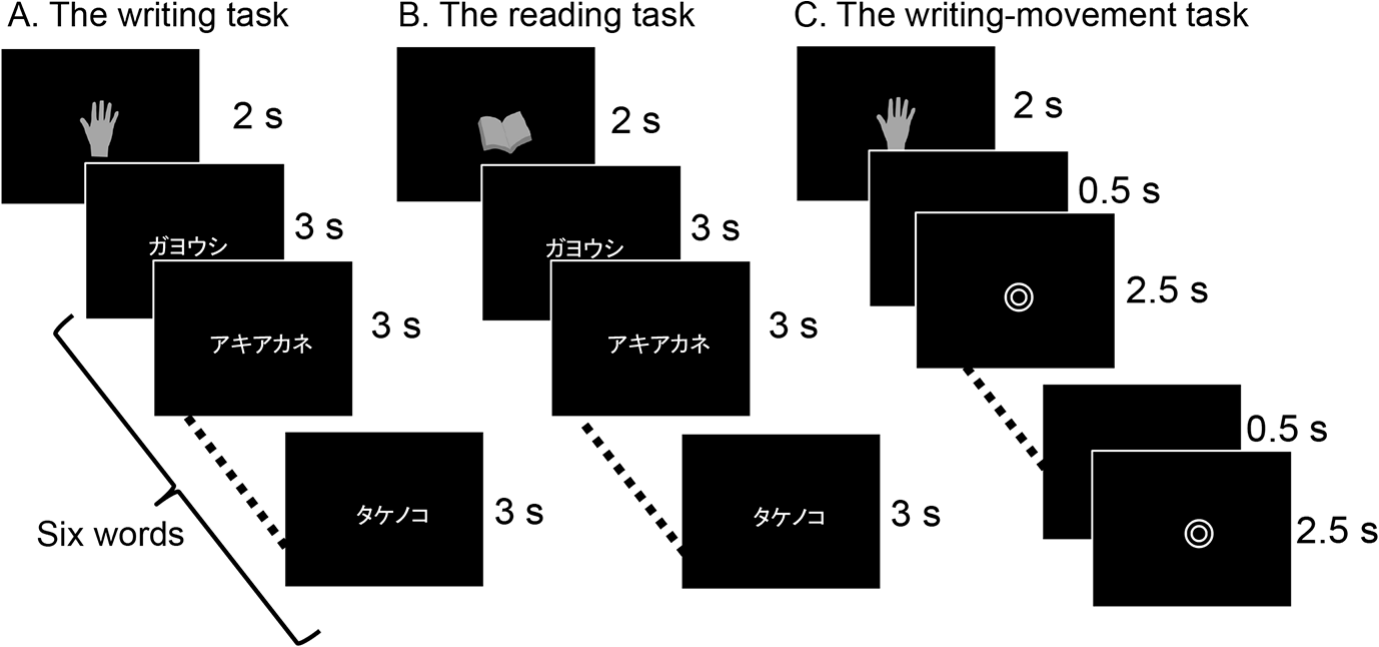
Design of the writing experiment. The writing experiment used an fMRI block design that was almost the same as the typing experiment. Each session consisted of three different tasks presented pseudo-randomly in five blocks of 20-s duration. In the writing-movement task (C), subjects were instructed to move their right index finger randomly when the double circle symbol was present on the monitor. Subjects had practiced this task before the scan. fMRI: functional magnetic resonance imaging.

Two conditions, each consisting of three tasks, were designed for the fMRI scan paradigm: the typing condition and the writing condition. The typing condition consisted of the typing task, the reading task, and the typing-movement task. The writing condition consisted of the writing task, the reading task, and the writing-movement task. Tasks were performed across six scanning sessions: three sessions for the typing condition and three sessions for the writing condition. Typing and writing sessions were alternated. Before each session, subjects were instructed on condition they were going to perform. In each session, five blocks of stimuli were pseudo-randomly presented: two writing or typing tasks, two movement tasks, and one reading task (see details below). The order of sessions was varied across subjects. A summary of each experiment is presented in Figs 2 and 3.

We adopted and modified a paradigm originally employed by Katanoda et al. for the writing and typing tasks [13]. In this paradigm, it is assumed that the process of typing or writing can be divided into three cognitive components, as mentioned in the Introduction (Fig 1). The “typing > reading” contrast was assumed to represent processes (2-T) and (3), and the “typing > typing-movement” contrast was assumed to represent processes (1) and (2-T). Therefore, the process we were most interested in for typewriting, i.e., (2-T), was calculated by the conjunction analysis of the “typing > reading” and “typing > typing-movement” contrasts. In the same manner, the conjunction analysis of the “writing > reading” and “writing > writing-movement” contrasts was expected to reveal the neural substrates of process (2-W) that are crucial for the processing of writing (Fig 1C).

Prior to participation in the actual experiment, each subject practiced the keyboard typing task and the writing task while lying down to ensure that they could perform the tasks. Subjects also practiced the movement tasks to ensure that they had similar timing, spatial range, speed, and use of both hands as in the typing and writing tasks. We instructed subjects not to type or write actual words or simple bigrams in the movement tasks. During this practice session, finger movements or typed outputs for each task were checked by eye to confirm that the subject performed the tasks almost perfectly. We recorded which hand was used to press each key to calculate the difference between the right hand and the left hand usage in the actual typewriting tasks.

### Typing condition

In each fMRI session for the typing condition, we presented five pseudo-randomly ordered 20-s blocks of stimuli that consisted of the typing task (n = 2 blocks), the reading task (n = 1 block), and the typing-movement task (n = 2 blocks). Between each block and at the end of the fifth block, a fixation cross appeared for 15 s to signify a period of rest. This 15-s fixation period occurred six times in each fMRI session. Subjects were instructed to keep their hands on the home keys (“j” and “f”) when at rest. Home keys were identifiable to the touch by a small bump on the keys. We took great care to ensure that subjects were in a comfortable typing position prior to the start of scanning. The keyboard was positioned on the subject’s waist. Head movement was minimized by placing small foam cushions on each side of the subject’s head. Subjects were instructed not to correct any mistakes that were made during scan sessions.

#### 1. Typing task

**Fig 4:**
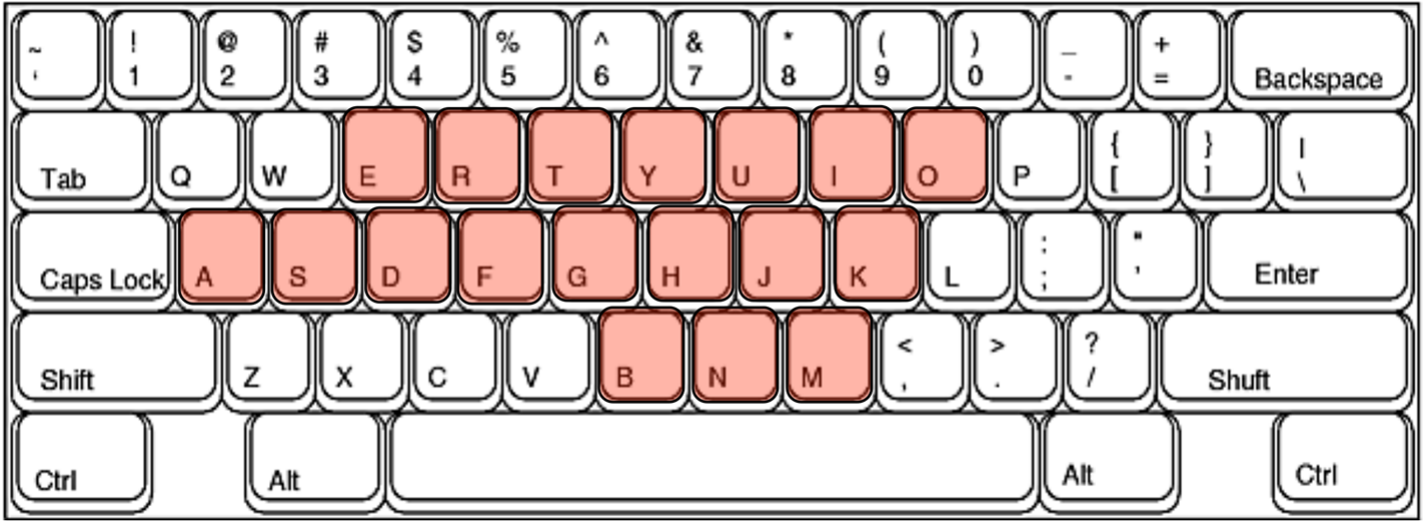
Positions of the keys required to type the words included in the word list. The typing task required the subjects to type the letters corresponding to the red colored keys. The keys q, w, z, x, c, v, p, and l were excluded because there are few opportunities to press these keys for Japanese typists and these keys are located in the corners of the QWERTY keyboard.

In the typing task, a word was presented on a screen and the subject was instructed to type the visually presented word on a keyboard (Fujitsu CP218230-02) that had been modified for use in the scanner by eliminating all ferro-magnetic components. The word list for the task consisted of 110 kana nouns obtained from the Nippon Telegraph and Telephone corporation (NTT) psycholinguistic database called "Lexical Properties of Japanese" [14]. Words were matched on linguistic parameters including word frequency, imagability, number of syllables, and key positions on the QWERTY keyboard so as not to require unequal hand usage. All words consisted of four or five kana characters (seven to nine alphabetic characters) and did not include the Roman characters q, w, z, x, c, v, p, or l, because there are relatively few opportunities to press these keys in the Japanese language and these characters are located in the corners of the QWERTY keyboard (Fig 4). No word was shown more than once in the experiment (i.e., the six scanning sessions containing the writing, reading, and the typing conditions).

Before the typing task, a picture of a hand, which indicated that the subject should type, was presented for 2000 ms followed by six different words presented sequentially over a period of 18000 ms with no interval and subjects were instructed to type each word on the keyboard (Fig 2A). We adopted the most popular way of typing Japanese called “alphabetical input”. In this method, a western-style keyboard is used, and the kana character “ka” is produced by typing “k” followed by “a” [1]. Although there are other ways of typing, all subjects were instructed to type using the alphabetical input method.

The keys pressed were recorded through the optical fibers placed in the keyboard buttons and the accuracy of typewriting was calculated offline. The percentage of left-hand and right-hand usage on the keyboard was calculated for each word to ensure that there was no bias in hand use. Behavioral data obtained from the typing experiment were processed in MATLAB 7.7.0 (MathWorks Inc., Natick, MA, USA). Accurate trials were defined as those for which subjects produced the correct key-pressing sequence for a given word. We were primarily interested in spelling errors as opposed to typing errors such as a slip in a keypress to the adjacent key, and we therefore did not consider key presses that were either one to the right or left of the correct key to be incorrect (e.g. for the letter “y”, “t” or “u” were considered correct). Typing speed (number of keys per second) was calculated offline to check the difference between consecutive key presses for each trial involving a motor control task.

#### 2. Reading task

For the reading task, a picture of a book, which indicated that the subject should read, was presented for 2000 ms followed by six different words presented sequentially over a period of 18000 ms with no interval, and subjects were instructed to read each word silently and not to move their fingers (Fig 2B). The word list for the reading task was the same as that used in the writing task, and no word was shown more than once in the experiment.

#### 3. Typing-movement task

In the typing-movement task, which was designed as a control, a double circle (◎) was presented for 2000-ms periods with a blank screen presented for 1000 ms between each period, and subjects were instructed to type randomly with both hands at a pre-learned speed during the presentation of the double circle (Fig 2C). Behavioral data were obtained in the same manner as for the typing task.

### Writing condition

In each fMRI session for the writing condition, we presented five pseudo-randomly ordered 20-s blocks of stimuli that consisted of the writing task (n = 2 blocks), the reading task (n = 1 block), and the writing-movement task (n = 2 blocks). Between each block and at the end of the fifth block, a fixation cross appeared for 15 s to signify a period of rest. This 15-s fixation period appeared six times in each fMRI session.

#### 1. Writing task

In the writing task, subjects were instructed to write words with their right index finger in the air. It is natural for Japanese people to write with the index finger in the air, and they often move their index finger to trace the imagined letter. Before the task, a picture of a hand, which indicated that the subject should write, was presented for 2000 ms followed by six different words presented sequentially over a period of 18000 ms with no interval and subjects were instructed to trace each word in the air with their index finger in Japanese phonograms (kana) (Fig 3A). The word list for this task was the same as that used in the typing condition. During the fMRI scan, finger movements were checked by examiners via a video monitor.

#### 2. Reading task

For the reading task, a picture of a book, which indicated that the subject should read, was presented for 2000 ms followed by six different words presented sequentially over a period of 18000 ms with no interval, and subjects were instructed to read each word silently (Fig 3B). The word list for the reading task was the same as that used in the typing condition.

#### 3. Writing-movement task

In the writing-movement task, a double circle (◎) was presented and subjects were instructed to move their index finger randomly at a pre-learned speed, in the way they do when they write in the air. The double circle was presented for 2500-ms periods with a blank screen presented for 500 ms between each period (Fig 3C). The stimulus duration in the writing-movement task was longer than that in the typing-movement task to control for the difference in the time taken to complete these two tasks. During the fMRI scan, finger movements were checked by examiners via a video monitor.

### Visual stimuli

Visual stimuli were presented on a screen mounted on a head coil through a projector (Victor, Japan, DLA-HD10KS, spatial resolution 1024 × 768 pixels, refresh rate 60 Hz) outside the scanner room. Pictures were grayscale images (Figs 2 and 3). Stimulus presentation was synchronized with the pulses of the fMRI scanner and was controlled by E-Prime software (http://www.pstnet.com/products/e-prime/) run on a Windows PC.

### Data acquisition

All MRI experiments were performed using a Tim-Trio MR scanner (Siemens, Erlangen, Germany) with a standard 12-channel head matrix coil operating at 3.0 Tesla. After initial localizer images had been obtained, T1-weighed anatomical images were obtained with an inversion recovery-prepared magnetization-prepared rapid acquisition with gradient echo (MPRAGE) sequence with a matrix size of 256 × 256 over a field of view of 256 mm, and a slice thickness of 1 mm. For functional imaging, we used single-shot gradient-echo echo planar imaging (EPI) with a repetition time of 2500 ms, echo time of 30 ms, flip angle of 90°, field of view of 240 mm, matrix size of 64 × 64, and slice thickness of 3.6 mm with no gap. Seventy-six slices parallel to the anterior commissure-posterior commissure line were acquired for each volume. We acquired 76 volumes in each of the six sessions, leading to a total of 456 volumes per subject.

### Image processing

Data analysis was carried out using SPM8 (available at http://www.fil.ion.ucl.ac.uk/spm/) implemented in MATLAB 7.7.0 (MathWorks Inc.) applying the general linear model [15]. The anatomical MPRAGE image was normalized to the standard Montreal Neurological Institute (MNI) reference anatomical template brain [16]. We corrected for head motion by realigning each scan to the first image, co-registering the functional scans with the anatomical MPRAGE scan, and then normalizing them via the same warping parameters used to normalize the MPRAGE scan. The images were then re-sliced to 2 × 2 × 2 mm^3^ and smoothed with an 8-mm full-width at half-maximum isotropic Gaussian kernel.

### fMRI data analysis

The first volume of EPI in each task was excluded from the statistical analyses because it contained the task instructions. The experimental conditions were modeled as box-car functions convolved with the hemodynamic response function with a time derivative. Global scaling was not used, high-pass temporal filtering with a cut-off of 128 s was applied, and serial autocorrelations were modeled with an autoregressive (AR 1) model in SPM8. Eleven contrasts were estimated for each subject: typing > fix, reading > fix, writing > fix, typing movement > fix, writing movement > fix, typing > reading, writing > reading, typing > typing movement, writing > writing movement, typing > writing, and writing > typing.

The second-level group study was based on a random effects model, to account for between-subject variance and to allow inference at the population level. The summary scans of the 16 subjects were compared using 11 one-sample *t*-tests, one for each of the 11 comparisons performed. Finally, we obtained 11 statistical maps for the group. We report the results obtained at *p* < 0.05, voxel-wise corrected for multiple comparisons using the family-wise error correction for the whole brain with a spatial extent larger than 30 voxels and cluster-level corrected at *p* < 0.05.

Areas of activation associated with typing were identified by examining the “typing > reading” contrast and “typing > typing movement” contrast. To exclude clusters in which typing activation was negative in relation to the baseline fixation condition, we applied a mask generated by the “typing > fix” contrast at a low threshold of uncorrected *p* < 0.05 to the activation maps generated in the “typing > reading” contrast and “typing > typing movement” contrast. For the same reason, we applied a mask generated by the “writing > fix” contrast at a low threshold of uncorrected *p* < 0.05 to the activation maps generated in the “writing > reading” contrast and the “writing > writing movement” contrast.

Next, we used a conjunction method to identify the regions that were the most crucial for typing and writing, namely processes (2-T) and (2-W) in Fig 1. The two conjunctions tested were: (a) “typing > reading” and “typing > typing movement”, and (b) “writing > reading” and “writing > writing movement”, using the statistical maps generated in the prior analyses.

Additionally, to elucidate the difference between the neural substrates for typing and writing, we compared the “typing > typing movement” contrast and the “writing > writing movement” contrast directly by constructing the (typing > typing movement) > (writing > writing movement) contrast and (writing > writing movement) > (typing > typing movement) contrast.

In summary, we tested six contrasts: typing > reading, writing > reading, typing > typing movement, writing > writing movement, (typing > typing movement) > (writing > writing movement), and (writing > writing movement) > (typing > typing movement), and two conjunctions: (a) typing > reading and typing > typing movement, (b) writing > reading and writing > writing movement (Fig 1C).

The coordinates of the statistically significant activations were expressed in MNI space and the anatomical localization of the statistical maps was determined with reference to the SPM Anatomy Toolbox Atlas [17] and the Anatomical Automatic Labeling atlas developed for MNI space [18]. Brodmann areas (BAs) were identified from the template developed for MRIcron.

## Results

### Behavioral data

The total number of keys pressed during the typing task was 278.1 (SD = 7.20), the difference between the right hand and the left hand usage (i.e.; the number of keys pressed by the right hand minus the number pressed by the left hand) was -4.75 (SD = 5.09), and the accuracy of typing was 96.34% (SD = 4.83%), indicating that all subjects achieved the typing tasks almost perfectly using both hands. The total number of keys pressed during the typing-movement task was 419.9 (SD = 86.8) and the difference between the right hand and the left hand usage was 5.31 (SD = 42.4). There was no linguistic structure or repeated bigrams in the output of the typing-movement task. The mean typing speed was 2.56 keys per second (SD = 1.20 keys per second) during the typing task, and 5.83 keys per second (SD = 0.07 keys per second) during the typing-movement task. The number and speed of keys pressed in the typing-movement task was therefore larger and faster, respectively, than that in the typing task. Due to these differences, no activation associated with writing movement and key pressing should be present in the subtracted contrasts.

### Group analysis of MRI data

**Fig 5:**
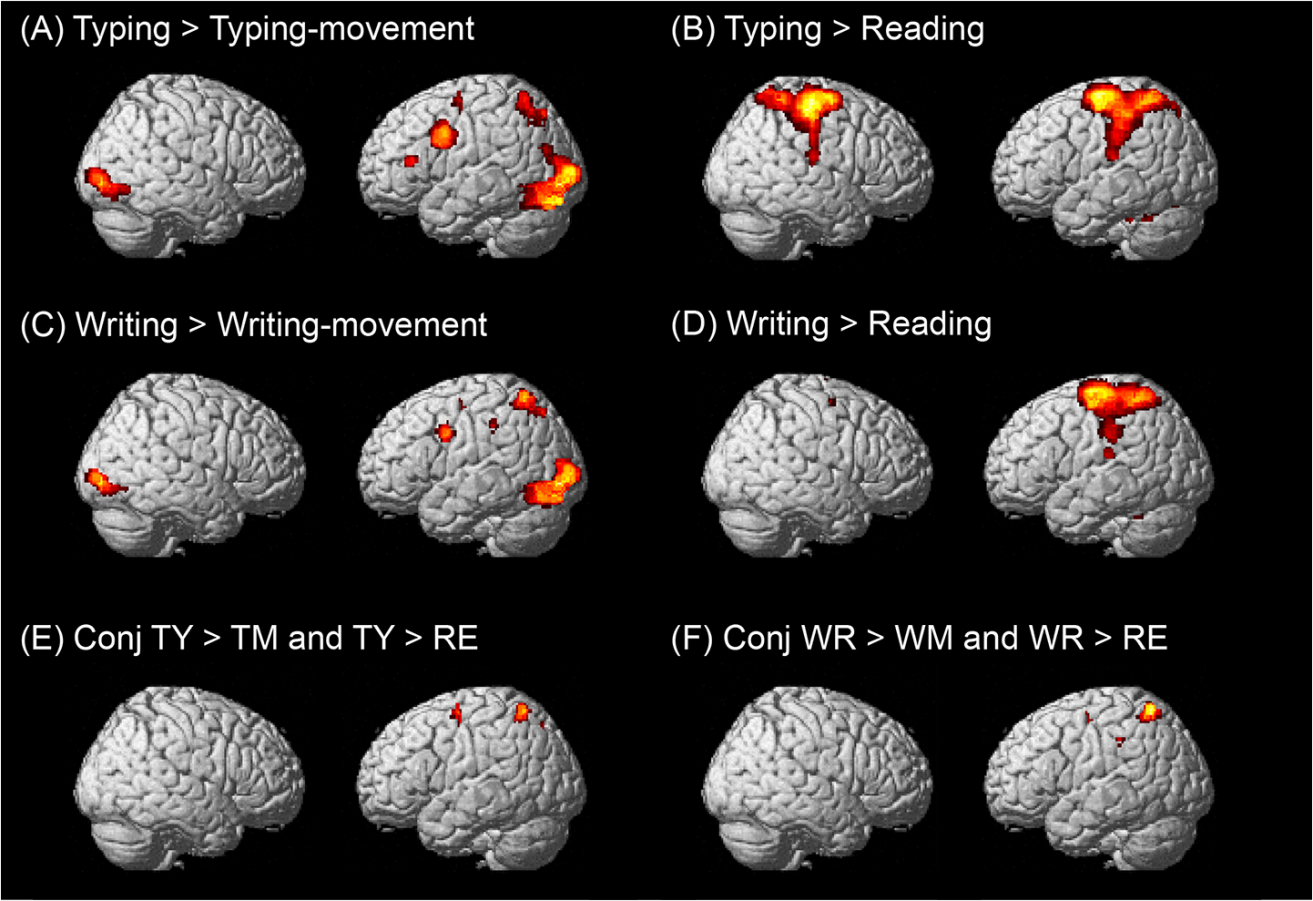
Brain areas activated in the typing and writing experiments. Each brain area was projected on a standard rendered SPM template brain. All maps were thresholded at a significance level of *p* < 0.05 voxel-wise corrected for multiple comparisons using family-wise error correction. (A) Typing > typing-movement contrast, (B) Typing > reading contrast, (C) Writing > writing-movement contrast, (D) Writing > reading contrast, (E) Conjunction of typing (TY) > typing movement (TM) contrast and typing (T) > reading (RE) contrast, (F) Conjunction of writing (WR) > writing movement (WM) contrast and writing (WR) > reading (RE) contrast.

The results of the random effects group analysis are summarized in Tables 1–5 and Fig 5. The results of the “typing > typing movement” contrast are shown in Table 1 and Fig 5A. Several activated areas were present including the bilateral inferior occipital gyri extending to the left inferior temporal cortex, the left frontoparietal areas such as the SPL, the posterior part of the left MFG, the upper and lower parts of the left pre-central gyrus, and the left IFG, corresponding to Broca’s area.

**Table 1:** The local maxima of activated regions in the typing > typing-movement contrast.

Activated brain region	Approximate BA	MNI coordinate			Z-score
		x	y	z	
Left occipital	IOG (BA 18)	-22	-92	-4	>8
		-36	-80	-10	>8
Left parietal	SPL (BA 7A)	-26	-60	44	>8
Right occipital	IOG (BA 18)	30	-88	-6	>8
Left frontal	PrCG/MFG (BA 6)	-48	6	34	7.68
	PrCG (BA 6)	-28	-6	58	5.37
	IFG (BA 45)	-46	28	14	5.82
Right cerebellum	Lobule VI (Hem)	8	-72	-22	5.72

Significance level was set at *p* < 0.05 (family-wise error).

BA: Brodmann area, MNI: Montreal Neurological Institute, IOG: inferior occipital gyrus, SPL: superior parietal lobule, PrCG: pre-central gyrus, MFG: middle frontal gyrus, IFG: inferior frontal gyrus, Hem: hemisphere.

**Table 2:** The local maxima of activated regions in the typing > reading contrast.

Activated brain region	Approximate BA	MNI coordinate			Z-score
		x	y	z	
Left cerebellum	Lobule VI (vermis)	0	-64	-18	>8
		-26	-48	-26	>8
		-16	-52	-20	>8
		-8	-26	-18	5.66
Left frontal	PrCG (BA 6)	-28	-10	62	>8
		-34	-16	62	>8
Left parietal	SPL (BA 7)	-30	-58	38	5.10
Right frontal	PrCG (BA 4)	36	-24	56	>8
	mCingG (BA 6)	6	-26	44	5.06
Right sub-lobar	Thalamus	18	-22	14	6.88
Left sub-lobar	Thalamus	-12	-22	18	6.57

Significance level was set at *p* < 0.05 (family-wise error).

BA: Brodmann area, MNI: Montreal Neurological Institute, PrCG: pre-central gyrus, SPL: superior parietal lobule, mCingG: middle cingulate gyrus.

**Table 3:** The local maxima of activated regions in the writing > writing-movement contrast.

Activated brain region	Approximate BA	MNI coordinate			Z-score
		x	y	z	
Left occipital	IOG (BA 18)	-20	-92	-4	>8
		-36	-80	-10	>8
	IOG (BA 19)	-40	-68	-10	7.68
Right occipital	IOG (BA 18)	30	-88	-6	7.80
Left parietal	SPL (BA 7)	-28	-56	56	6.78
		-24	-60	62	6.37
	SMG (BA 40)	-42	-36	40	5.21
Right parietal	SPL (BA 7)	-20	-68	46	6.64
Left frontal	PrCG/MFG (BA 6)	-48	4	34	5.90
	MFG (BA 6)	-22	-12	50	5.48

Significance level was set at *p* < 0.05 (family-wise error).

BA: Brodmann area, MNI: Montreal Neurological Institute, IOG: inferior occipital gyrus, SPL: superior parietal lobule, SMG: supramarginal gyrus, PrCG: pre-central gyrus, MFG: middle frontal gyrus.

**Table 4:** The local maxima of activated regions in the writing > reading contrast.

Activated brain region	Approximate BA	MNI coordinate			Z-score
		x	y	z	
Right cerebellum	Lobule V (vermis)	14	-54	-18	>8
	Lobule VI (Hem)	4	-64	-18	>8
		24	-46	-26	7.81
Left frontal	PrCG (BA 4)	-32	-26	58	>8
	PrCG (BA 6)	-24	-16	68	7.46
	SFG (BA 6)	-26	-10	60	>8
Left sub-lobar	Thalamus	-16	-24	8	7.69
		-6	-26	-8	4.79
	Midbrain	-2	-30	-14	4.95
Left cerebellum	Lobule VI (Hem)	-28	-46	-24	5.85
		-24	-54	-20	5.19
Right frontal	SFG (BA 6)	22	-10	56	5.83
Left parietal	SMG (BA 40)	-50	-26	20	5.64
Right sub-lobar	Thalamus	18	-22	14	5.38

Significance level was set at *p* < 0.05 (family-wise error).

BA: Brodmann area, MNI: Montreal Neurological Institute, Hem: hemisphere, PrCG: pre-central gyrus, SFG: superior frontal gyrus, SMG: supramarginal gyrus.

**Table 5:** The local maxima of activated regions in the conjunction analysis.

Activated brain region	Approximate BA	MNI coordinate			Z-score
		x	y	z	
**Conjunction Typing > Reading and Typing > Typing movement**					
Left parietal	SPL (BA 7)	-28	-58	60	5.91
		-14	-70	50	4.94
		-30	-44	46	4.89
	IPS/SPL (BA 7)	-30	-60	38	5.05
Right cerebellum		8	-72	-22	5.72
Left frontal	MFG/SFG (BA 6)	-28	-6	58	5.37
**Conjunction Writing > Reading and Writing > Writing movement**					
Left parietal	SPL (BA 7)	-26	-58	60	6.47
	SMG (BA 40)	-42	-34	38	5.08
Left frontal	MFG/SFG (BA 6)	-22	-12	50	5.48

Significance level was set at *p* < 0.05 (family-wise error).

BA: Brodmann area, MNI: Montreal Neurological Institute, SPL: superior parietal lobule, IPS: intraparietal sulcus, MFG: middle frontal gyrus, SFG: superior frontal gyrus, SMG: supramarginal gyrus.

The results of the “typing > reading” contrast are shown in Table 2 and Fig 5B. Large extents of the bilateral frontoparietal regions were significantly activated. Activated regions included the bilateral pre-central gyrus, the left SPL, and the right middle cingulate gyrus. The left cerebellum, including the left vermis, and the left hemisphere were also significantly activated. Subcortical activation was observed in the bilateral thalamus.

The regions that demonstrated significant activation for the “writing > writing movement” contrast are shown in Table 3 and Fig 5C. The activated areas were the bilateral inferior occipital gyri spreading into the left inferior temporal cortex, the left frontoparietal areas including the left SPL and the SMG, the left pre-central gyrus, and the posterior end of the left MFG. The right SPL was also activated, but cerebellum activation was not observed.

The regions that demonstrated significant activation for the “writing > reading” contrast are shown in Table 4 and Fig 5D. Activation in the cerebral cortex showed strong left hemispheric lateralization. The following activation peaks were observed in the left frontoparietal cortical regions: the posterior end of the left SFG extending to the left MFG and the left SPL and the left anterior limb of the left SMG. The bilateral cerebellum, the posterior part of the right SFG, and the right thalamus were also activated.

The regions that demonstrated significant activation for the “typing > writing” contrast are shown in the Supporting Information (S1 Fig, S1 Table). The results of the “writing > typing” contrast showed no activated brain regions.

The results of the conjunction analysis of the “typing > typing movement” and “typing > reading” contrasts are shown in Table 5 and Fig 5E. The main cerebral activation was observed in two regions in the left hemisphere: the anterior portion of the SPL extending to the SMG and the posterior part of the MFG/SFG. Activation in the posteromedial part of the left intraparietal sulcus (IPS) just superior to the angular gyrus (AG) and the right cerebellum was also observed. The results of the conjunction analysis of the “writing > writing movement” and “writing > reading” contrasts are shown in Table 5 and Fig 5F. Three left frontoparietal regions were significantly activated: the left anterior SPL, the posterior part of the left MFG/SFG, and the left SMG. The posterior parts of the MFG activated in both conjunction contrasts correspond to Exner’s area.

**Fig 6:**
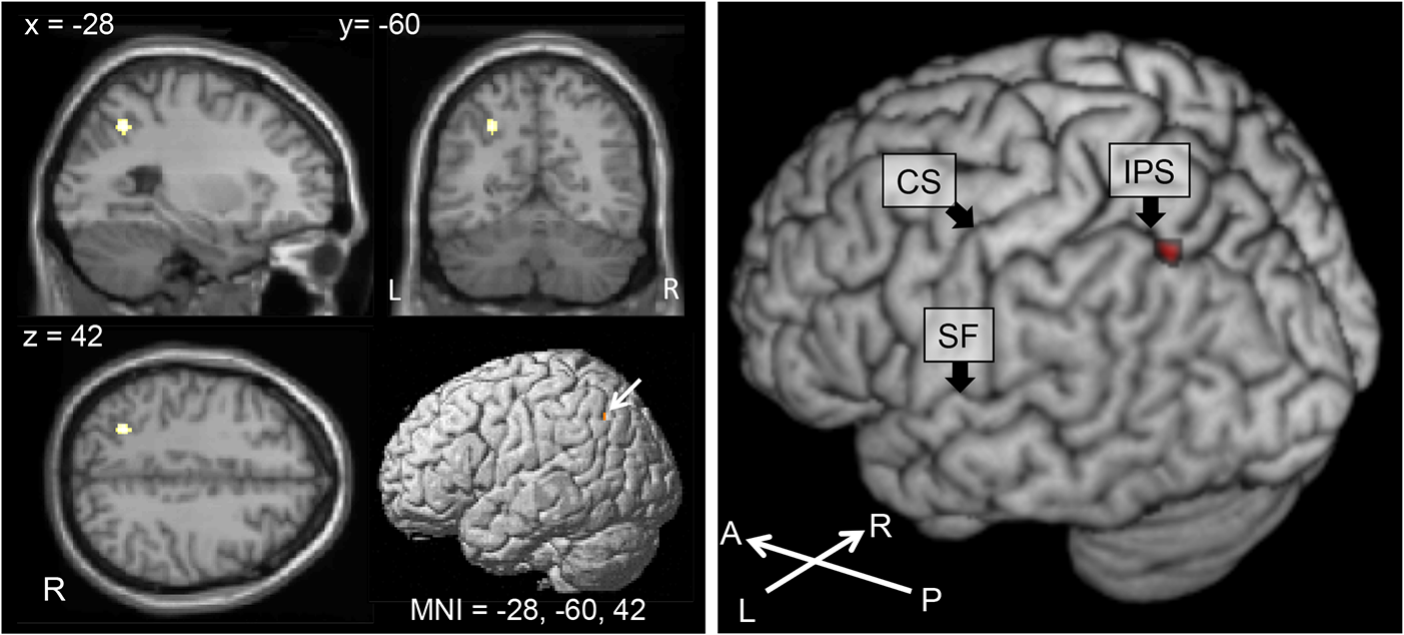
Brain areas activated in the (TY >TM) > (WR > WM) contrast. (A) The location of the IPS/SPL activations obtained from the (TY >TM) > (WR > WM) contrast. The map was thresholded at a significance level of *p* < 0.05 voxel-wise corrected for multiple comparisons using family-wise error correction. Significant activation was observed in the posterior portion of the left medial intraparietal cortex (MNI peak: -28, -60, 42; Z score = 5.32). (B) The (TY > TM) > (WR > WM) contrast activity was superimposed on a 3D rendering of a human brain. Significant activation was observed in the posterior portion of the left medial intraparietal cortex (red). CS: central sulcus, IPS: intraparietal sulcus, SF: Sylvian fissure, A: anterior, P: posterior, L: left, R: right, TY: typing, TM: typing-movement, WR: writing, WM: writing-movement.

The results of the (typing > typing movement) > (writing > writing movement) contrast are shown in Fig 6. There was significant activation in the left posteromedial IPS. The results of the (writing > writing movement) > (typing > typing movement) contrast showed no activated brain regions.

## Discussion

Keyboard typing is a complex perceptual, cognitive, and motor process that involves the generation of motor commands that are not directly related to letter shape [11]. Because of this difference from writing and the existence of dystypia, an isolated typing disorder, we hypothesized that there would be some differences in the neural networks for typewriting and handwriting, especially in the peripheral transition processes.

Our fMRI study revealed three brain regions activated during both tasks: the left anterior SPL, the left SMG, and the posterior end of the left MFG/SFG. These brain regions, known as the “writing centers” from numerous lesion studies and neuroimaging studies, were activated in both the typewriting and the writing task. Additionally, we identified a typing-specific brain region: the left posteromedial IPS. To the best of our knowledge, this is the first fMRI study to compare the neural basis of Japanese keyboard typing and writing directly in the same individuals and to focus on the left frontoparietal brain regions.

### The left SPL

The conjunction between the “typing > typing movement” and “typing > reading” contrasts showed peak activation in the anterior part of the left SPL (MNI peak: -28, -58, 60) and the conjunction between the “writing > writing movement” and “writing > reading” contrasts showed almost the same activation region (MNI peak: -26, -58, 60).

The left SPL is responsible for sequence production in written language [19–21]. Damage to the left SPL is associated with apraxic agraphia, an impairment in writing in which the orthographic production of letters and words is abnormal despite normal sensorimotor function and word and letter knowledge [20, 22]. fMRI studies have reported left SPL activation during writing [6, 13, 21, 23] and a meta-analysis of fMRI studies of the brain region responsible for English written word production indicated a left anterior SPL/IPL cluster that was associated with the peripheral but not central processes of written word production (MNI peak: -36, -40, 57) [7]. Katanoda et al. and Sugihara et al. used fMRI to examine the neural substrates for writing Japanese kana characters and also reported activation of the left SPL (MNI peak: -32, -38, 56 and -36, -42, 48) [13, 23]. These data indicate that the left SPL is an essential brain region for writing both English alphabetic words and Japanese kana characters.

In addition, our data suggest the same brain region, the left SPL, was also activated in the typewriting task. These results suggest that the left SPL is not only the writing center, but also the typing center. Although no lesion studies have implicated the left SPL in dystypia, Magrassi et al. reported a patient who exhibited handwritten and typed production errors, but no errors in oral spelling, after electrical stimulation of a restricted area of the SPL [24]. Recent functional imaging studies have made the same proposal. For example, Gordon et al. used fMRI to examine brain activation during the production of typing motor sequences and indicated activation of the SPL [8], and Purcell et al. investigated the neural basis of spelling during keyboard typing and reported fMRI activation of clusters in the left SPL (MNI peak: -28, -60, 46). These previous data and our results strongly suggest that activation of this region is involved in both writing and typing. The left SPL has also been associated with the planning or generation of complex sequential movements regardless of writing [25] and the integration of the body schema with ego-centric frames of reference [26]. In this respect, it is also possible that the activation of this area was the consequence of complex sequential finger movements, regardless of writing or typing processes. However, our results demonstrating the important role of the left SPL were drawn from subtraction of the motor execution process using control tasks.

### The left SMG

The conjunction between the “typing > typing movement” and “typing > reading” contrasts and the conjunction between the “writing > writing movement” and “writing > reading” contrasts showed activation in the left SMG. The left SMG has been shown by numerous neuropsychological studies to be important for writing, especially phoneme-grapheme conversion. Lesions in the left SMG have been associated with phonological agraphia, which is defined as a loss of ability to write using phoneme-grapheme conversion [19, 27, 28]. Impaired spelling of pronounceable pseudowords is a characteristic of phonological agraphia. SMG lesion has been reported in patients with pure agraphia for kana (Japanese phonograms), which is characterized by errors in literal paragraphia (substitutions, additions, and omissions of letters) [29]. Despite a large number of studies of patients with left SMG lesions, there are no functional neuroimaging studies that have reported involvement of the left SMG in alphabet writing [7]. In this study we employed Japanese phonograms (kana), which are known to rely heavily on phoneme-grapheme conversion, rather than kanji or alphabetic words, as mentioned in the Introduction. In a previous neuroimaging study, the left SMG was active in a kana-writing task that placed a strong demand on phoneme-grapheme conversion [13, 23]. With regard to typewriting, we found significant activation in the left SMG extended from the left SPL. Alphabetical input is the most popular way of Japanese keyboard typing, and was the method of keyboard typing that we employed in this study. To perform alphabetical input typing, the subject has to separate the phoneme into the corresponding vowel and consonant. For instance, the kana character ‘ka’ is produced by typing ‘k’ followed by ‘a’ [1]. We suggest that Japanese typewriting needs much greater phoneme-grapheme conversion than alphabetic writing, and this may explain the left SMG activation in the typing task.

### The left IPS

**Fig 7:**
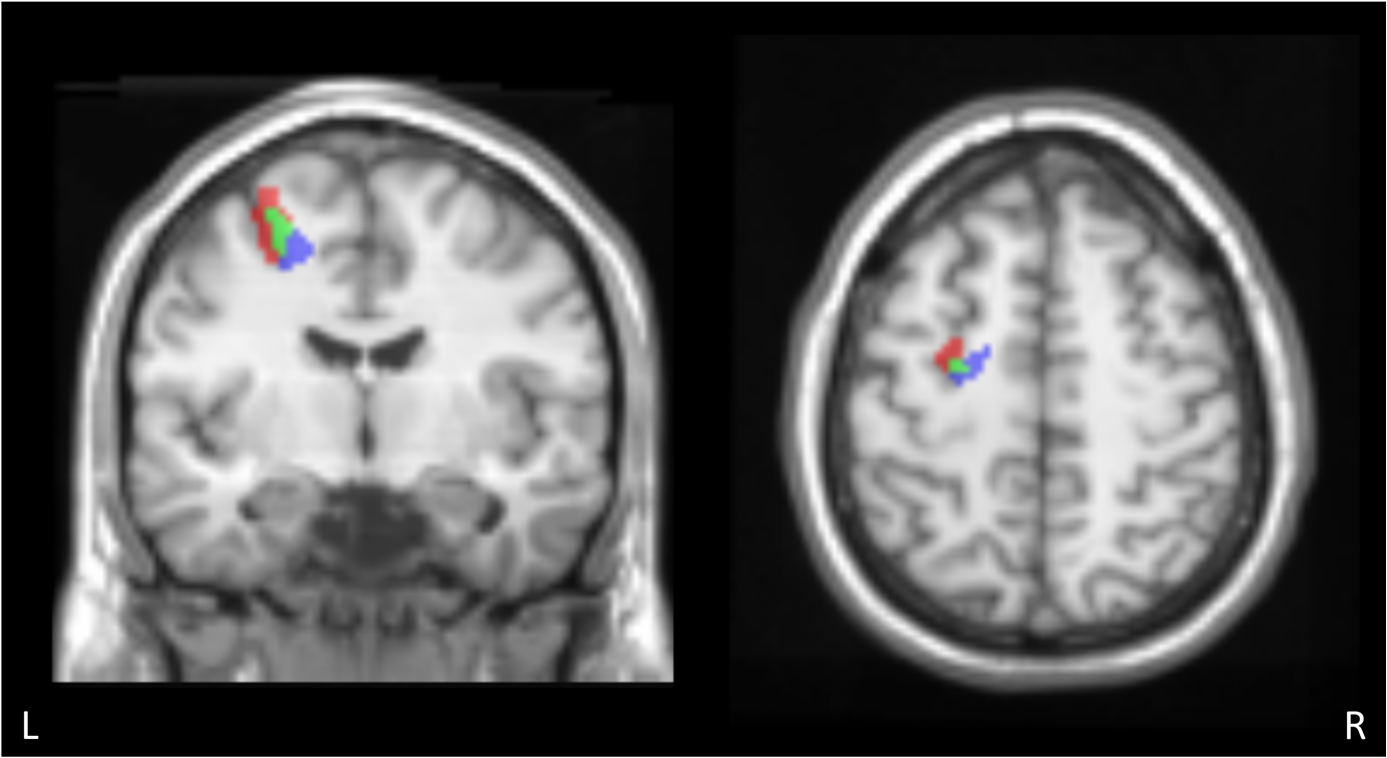
Overlap of the MFG activated regions obtained from the conjunction analysis of typing and writing. Overlap of the MFG activated regions obtained from the conjunction analysis of typing (red) and writing (blue). The green area represents the voxels that were identified in both contrasts. Overlap between two conjunctions was observed and the typing activity extended to dorsolateral and rostral region. MFG: middle frontal gyrus.

The conjunction between the “typing > typing movement” and “typing > reading” contrasts showed small peak activations in the left IPS just superior to the AG (MNI peak: -30, -60, 38), and the (typing > typing movement) > (writing > writing movement) contrast revealed significant activation in the left posteromedial part of the IPS (MNI peak: -28, -60, 42). These results indicated that these IPS regions were activated to a greater degree in the typing task than in the writing task (Fig 7). A meta-analysis of fMRI studies for English written word production that contained a typewriting task indicated that the posterior part of the IPS cluster was activated during central spelling processes, and this is very close to our activated area for typewriting (MNI peak: -30, -60, 46) [7]. Lesion studies have revealed that damage to the left IPS/SPL sparing the AG can result in spelling deficits associated with orthographic working memory [30]. From this point of view, our results could suggest higher orthographic working memory demands in typewriting than in handwriting. It is also possible that these results reflect the difference in the grapheme-motor command conversion process related to orthographic working memory. On the other hand, it is reasonable to suppose that handwriting requires more online working memory resources, because it takes longer. The lack of behavioral data prevents us from making a conclusion and further study is needed to make a comparison of the working memory demands between these two modalities.

In addition to such processes, another difference between typing and writing is the properties of the tools used, i.e., the keyboard vs. the pen. The keyboard places demands on highly specific space representations and on sequential on-line control of key-stroke actions. Traditionally, the left parietal cortex is considered as a major region responsible for actual tool use, and damage to this region is known to be associated with apraxia. Recent neuropsychological and neuroimaging data suggest that parietal regions, that is, the dorsal and medial parietal cortex, contribute to on-line monitoring/adjusting of ongoing prehensile action for tools [31]. Grefkes et al. proposed that the medial IPS was also crucial for transforming visual coordinates into motor programs and was activated bilaterally in the online control of goal-directed precision movements [32, 33]. It is interesting that only the left IPS was activated in our study. It is possible that our left-dependent activation is due to methodological differences between our study and that of Grefkes et al., as typewriting can be regarded as requiring language-specific visuomotor coordination of hand movements associated with the dominant hemisphere. Nevertheless, there might be another explanation for the laterality. A recent functional imaging study demonstrated that the bilateral IPS was significantly activated during mental rotation tasks known to be associated with visual representation [34]. Voyer et al. revealed that the functional lateralization of mental rotation processes was modulated by the skill with which the task was performed. With increasing practice there was a shift from a right hemisphere dominance to a left hemisphere dominance [35]. In accordance with their proposal, it is possible that left IPS activation during the typing task was due to the keyboard property as an over-learned visuospatial association tool.

Our results showed no activation of the left AG. Dejerine’s classic study and subsequent lesion studies support the role of the left AG as a center of lexical processing that underlies both spelling and reading [19, 36, 37]. In this respect, it is reasonable that our conjunction analyses for both typing and writing did not reveal any activation in the left AG, because our experiment aimed to elucidate the peripheral processes and placed very few demands on central processes such as semantic and lexical processes, as described in the Introduction.

### The left MFG/SFG

We identified significant activation associated with both the typing and the writing task in the left MFG/SFG, corresponding to the premotor cortex. The conjunction between the “typing > typing movement” and “typing > reading” contrasts showed peak activation in the posterior part of the MFG/SFG (MNI peak: -28, -6, 58), and the conjunction between the “writing > writing movement” and “writing > reading” contrasts showed activation ventromedial and caudal to that of the typing conjunction contrast (MNI peak: -22, -12, 50) (Fig 7).

Patients with lesions in the left MFG and IFG, known as Exner’s area [38, 39], experience writing disturbances characterized by the omission of words in a sentence or badly written words. Japanese patients with pure agraphia due to damage to Exner’s area are frequently reported to have a kana-dominant writing impairment including paragraphia [40, 41]. These lesion studies have led to the left dorsal premotor cortex being considered relevant to writing-specific processes that involve the generation of graphemic motor commands or a graphemic buffer for writing. The graphemic buffer is a working memory component of the spelling system that temporarily holds the sequence of graphemes during production of letter names for oral spelling or letter shapes for written spelling. Cloutman et al. studied stroke patients using magnetic resonance diffusion-weighted imaging and perfusion-weighted imaging to identify neuroanatomical regions associated with deficits to the graphemic buffer [42]. They found that the voxels most strongly associated with the graphemic buffer were in the pre-central and premotor cortex, involving BA 4 and 6, and the post-central gyrus, involving BA 2 and 3. Rapp and Dufor reported that the left superior frontal sulcus (MNI peak: -13, -11, 51) was the word-length-sensitive neural substrate in spelling [9] and hypothesized that this region was involved in orthographic working memory (i.e., the graphemic buffer) rather than the generation of graphemic motor commands. However, Roux et al. named this area the “Graphemic/Motor Frontal Area” [43]. They conducted a direct cortical stimulation and fMRI study and proposed that the area might correspond to the neural counterpart of the interface between the orthographic or graphemic abstract representations and the generation of motor commands for handwriting [43, 44].

Our data, however, revealed significant activation of the same brain regions during the typewriting tasks, indicating that this brain region was crucial not only for writing, but also for typing. Although it is difficult to clarify the actual role of the left MFG/SMG from our data, it is possible that the typed spelling was held in the graphemic buffer, or that the conversion of orthographic representations into typing-motor representations was produced in this area. Interestingly, a recent study has revealed that this region also plays a significant role in graphemic motor commands for typed production [5].

It is noteworthy that a direct comparison between the two left MFG/SFG activated clusters obtained from typing and writing conjunctions revealed a small gap (Fig 7). The activation observed in the typing conjunction was located dorsolateral and rostral to that observed in the writing conjunction, and only the rostral region had stronger activation in the typing task than in the writing task (Fig 7, red region). Although a direct comparison between the typing and writing revealed no significant difference in this area, it is possible that the left MFG/SFG is used in both modalities, but in different ways. The rostral premotor areas contain a greater proportion of neurons with sensory properties, whereas caudal areas contain a greater proportion with motor properties [45, 46]. Moreover, Simon et al. suggested that the region of the dorsal premotor area activated for motor preparation is located caudally and that activated for spatial attention and memory is located at a more rostral site [47]. Therefore, the more rostral activation in typing found in the present study might be due to greater sensory demand or spatial attention in typewriting than in writing. However, further study is needed to determine the difference related to this area.

### Crucial brain lesions for dystypia

Our study revealed a brain region that was dominant in typewriting over writing: the left posteromedial portion of the left IPS. In addition, activity in the left MFG/SFG was more rostral in the typing task than in the writing task. Therefore, we hypothesize that brain lesions in these areas could result in dystypia. The dystypia case reported by Ryu et al. had lesions in the bilateral border-zone regions, predominantly the left dorsal frontal area, that were relatively close to our left MFG/SFG activated area [2]. The patient reported by Cooks et al. had a lesion in the left temporoparietal region, which might have contained the left IPS [3]. These two case reports are therefore concordant with our hypothesis. However, the patient reported by Otsuki et al. had a lesion in the left frontal lobe that involved the foot of the second frontal convolution and frontal operculum, which seems to be lower than our left MFG activation [1]. Of course, it is difficult to draw conclusions on the brain regions critical for dystypia using only these three cases, and there might be several reasons for the inconsistency between the previous case reports and our results. First, the inconsistency may be due to individual anatomical variability in the typing and writing centers. Sugihara et al. reported inter-individual variability in the location of the writing center in the lower part of the anterior limb of the left SMG and other brain regions in the right hemisphere [23]. Hence, it is possible that the typing center in left lower part of the MFG/IFG was not consistent across individuals. Furthermore, the way in which subjects had learned to touch type is likely more variable than the way they learned to write, because writing, but not typing, must be mastered during compulsory education in Japan. This might result in further inter-individual variability. Second, although the patients from Ryu et al. and Cook et al. were able to type words without visual assistance, i.e.; touch type, similar to our subjects, the patient described by Otsuki et al. did not adopt the touch-typing technique. However, further studies need to be carried out to determine whether there is a difference in the neural substrate for non-touch typing and touch typing. In addition, further case reports are necessary to identify the functional networks in the brain that are involved in typing and writing.

## Conclusions

The current study examined the neural substrate for typing and compared it with that of writing in the same subjects. First, using a conjunction analysis, we identified the following three areas as crucial for the neural process of typing and writing: (1) the anterior part of the left SPL, (2) the left SMG, and (3) the posterior part of the left MFG/SFG, near Exner's area. These results demonstrate that the brain regions known as the “writing center” were also the neural center for typing. Second, a direct comparison between typing and writing revealed a typing-specific brain region: the left posteromedial IPS. In addition, activity in the left MFG/SFG was more rostral in the typing task than in the writing task. These findings provide the first evidence that the brain activity involved in Japanese typing is different from that involved in writing.

## Supporting Information

S1 Fig Brain areas activated in the typing > writing contrast. Each brain area was projected on a standard rendered SPM template brain. The map was thresholded at a significance level of *p* < 0.05 voxel-wise corrected for multiple comparisons using family-wise error correction. A large activation was observed in the right frontoparietal cortical region, i.e., the pre-central gyrus extending to the post-central gyrus. Activations were also observed in the following areas: left cerebellum, left post-central gyrus, right parietal operculum. The results of the writing > typing contrast showed no activated brain regions.
(TIF)

S1 Table The local maxima of activated regions in the typing > writing contrast. Significance level was set at *p* < 0.05 (family-wise error). BA: Brodmann area, MNI: Montreal Neurological Institute, PrCG: pre-central gyrus, PoCG: post-central gyrus.
(DOCX)
