# Correction: The Neural Basis of Typewriting: A Functional MRI Study

**DOI:** 10.1371/journal.pone.0137265

**Published:** 2015-08-28

**Authors:** Yuichi Higashiyama, Katsuhiko Takeda, Yoshiaki Someya, Yoshiyuki Kuroiwa, Fumiaki Tanaka

Fig 1 is an incorrect, previous version. Please view the corrected Fig 1 here.

**Fig 1:**
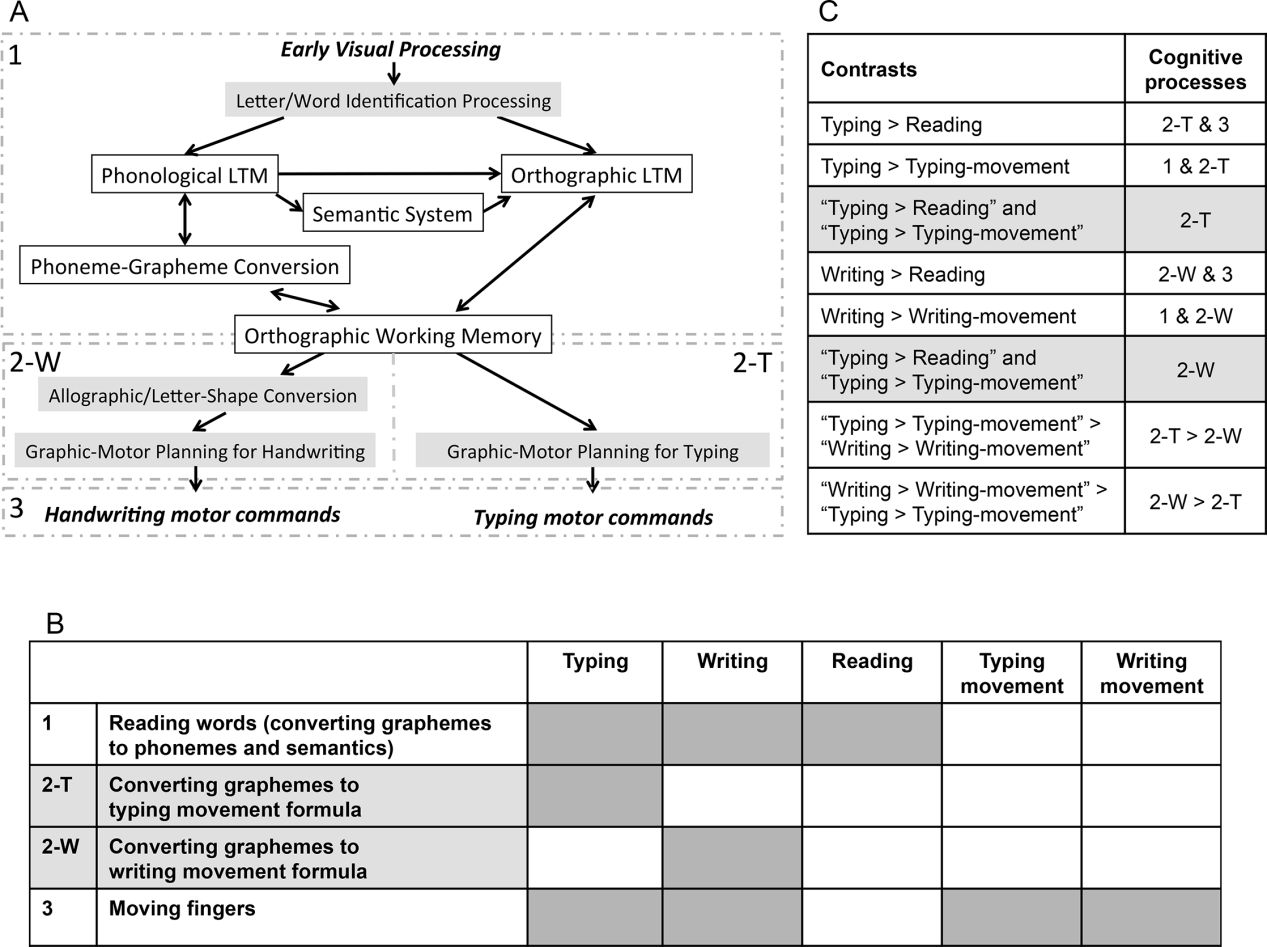
A diagram of the assumptions underlying the present analysis. **(A)** The cognitive model of handwriting and typing used in the present study. The labels 1, 2-W, 2-T, and 3 correspond to the labels in Fig 1B. **(B)** The simplified cognitive processes of typing and writing used in the present analysis. **(C)** The contrasts and corresponding cognitive processes. The labels 1, 2-W, 2-T, and 3 correspond to the labels in Fig 1B. Grey shading indicates conjunction contrasts.
