# Supplementary figures and images for: The Neural Basis of Typewriting: A Functional MRI Study
Source: PLoS One. 2015 Jul 28;10(7):e0134131. doi: 10.1371/journal.pone.0134131 (PMC4517759; doi:10.1371/journal.pone.0134131)

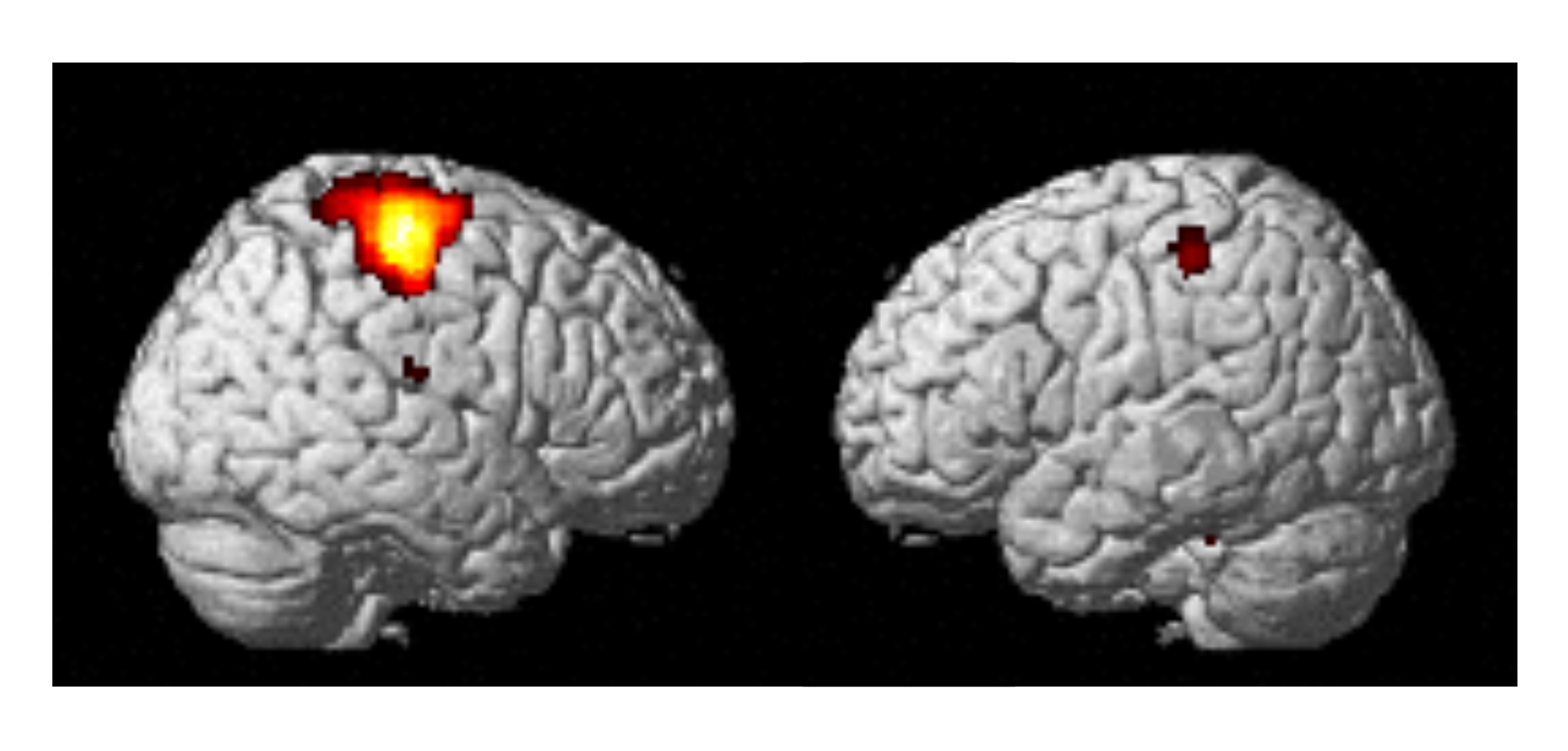

Supplement: S1 Fig — Each brain area was projected on a standard rendered SPM template brain. The map was thresholded at a significance level of p < 0.05 voxel-wise corrected for multiple comparisons using family-wise error correction. A large activation was observed in the right frontoparietal cortical region, i.e., the pre-central gyrus extending to the post-central gyrus. Activations were also observed in the following areas: left cerebellum, left post-central gyrus, right parietal operculum. The results of the writing > typing contrast showed no activated brain regions. (TIF) [file pone.0134131.s001.tif]
