# Supplementary material for: The Neural Basis of Typewriting: A Functional MRI Study
Source: PLoS One. 2015 Jul 28;10(7):e0134131. doi: 10.1371/journal.pone.0134131 (PMC4517759; doi:10.1371/journal.pone.0134131)
Supplement: S1 Table — Significance level was set at p < 0.05 (family-wise error). BA: Brodmann area, MNI: Montreal Neurological Institute, PrCG: pre-central gyrus, PoCG: post-central gyrus. (DOCX) [file pone.0134131.s002.docx]

| Activated brain region | Approximate BA | MNI coordinate | | | Z-score |
| --- | --- | --- | --- | --- | --- |
|  |  | x | y | z |  |
| Right frontal | PrCG (BA 4) | 38 | -24 | 54 | >8 |
| Right parietal | PoCG (BA 2) | 28 | -44 | 68 | 5.42 |
| Left cerebellum | Lobule V | -14 | -52 | -20 | >8 |
|  | Lobule V | -6 | -62 | -20 | 6.63 |
| Left parietal | PoCG (BA 1) | -46 | -30 | 56 | 5.88 |
| Right parietal | Parietal operculum | 44 | -22 | 22 | 5.57 |
